# Population Reference Values for Serum Methylmalonic Acid Concentrations and Its Relationship with Age, Sex, Race-Ethnicity, Supplement Use, Kidney Function and Serum Vitamin B12 in the Post-Folic Acid Fortification Period

**DOI:** 10.3390/nu10010074

**Published:** 2018-01-12

**Authors:** Vijay Ganji, Mohammad R. Kafai

**Affiliations:** 1 Human Nutrition Department, College of Health Sciences, Qatar University, P.O. Box 2713, Doha, Qatar; 2 Department of Mathematics, San Francisco State University, San Francisco, CA 94132, USA; kafai@sfsu.edu

**Keywords:** age, cobalamin, creatinine, MMA, methylmalonic acid, NHANES, National Health and Examination Survey, race-ethnicity, reference values, vitamin B-12

## Abstract

Serum methylmalonic acid (MMA) is elevated in vitamin B-12 deficiency and in kidney dysfunction. Population reference values for serum MMA concentrations in post-folic acid fortification period are lacking. Aims of this study were to report the population reference values for serum MMA and to evaluate the relation between serum MMA and sex, age, race-ethnicity, kidney dysfunction and vitamin B-12. We used data from three National Health and Nutrition Examination Surveys, 1999–2000, 2001–2002 and 2003–2004 conducted after folic acid fortification commenced (*n* = 18,569). Geometric mean MMA was ≈22.3% higher in non-Hispanic white compared to non-Hispanic black (141.2 vs. 115.5 nmol/L) and was ≈62.7% higher in >70 years old persons compared to 21–30 years old persons (196.9 vs. 121.0 nmol/L). Median serum MMA was ≈28.5% higher in the 1st the quartile of serum vitamin B-12 than in the 4th quartile of serum vitamin B-12 and was ≈35.8% higher in the 4th quartile of serum creatinine than in the 1st quartile of serum creatinine. Multivariate-adjusted serum MMA concentration was significantly associated with race-ethnicity (*p* < 0.001) and age (*p* < 0.001) but not with sex (*p* = 0.057). In this large US population based study, serum MMA concentrations presented here reflect the post-folic acid fortification scenario. Serum MMA concentrations begin to rise at the age of 18–20 years and continue to rise afterwards. Age-related increase in serum MMA concentration is likely to be due to a concomitant decline in kidney function and vitamin B-12 status.

## 1. Background

Serum methylmalonic acid (MMA) concentrations are elevated in vitamin B-12 deficiency. It is derived from l-methylmalonyl CoA due to impaired function of l-methylmalonyl CoA mutase [1]. l-methylmalonyl CoA mutase requires adenosylcobalamin, a coenzyme of vitamin B-12, which converts l-methlymalonyl CoA to succinyl CoA [2,3]. MMA interferes with energy production in mitochondria by inhibiting succinate dehydrogenase, an electron transport protein complex II [4]. Thus, the elevated MMA may have negative effect on health. It has been reported that elevated MMA may be detrimental to the nervous system and kidney [5].

Although serum vitamin B-12 concentrations are used to assess the nutritional status of vitamin B-12 [6], serum vitamin B-12 may not reflect true vitamin B-12 status because some individuals with low-normal serum vitamin B-12 exhibit tissue vitamin B-12 deficiency [7]. Vitamin B-12 circulates bound to holotranscobalamin (holoTC) (6–20%) and haptocorrin. Only the vitamin B-12 attached to holoTC, a biologically active vitamin B-12, is capable of delivering vitamin B-12 to the tissues. Although separate biological assay is available for measuring the holoTC, commonly used vitamin B-12 assays measures the total vitamin B-12 (holoTC B12 and haptocorrin B12). Because of this limitation, serum MMA is regarded as a reliable marker of vitamin B-12 deficiency in those with normal renal function [7].

Morris et al. [8], using data collected in the pre-folic acid fortification period (1991–1994) reported a ≈20% prevalence of high serum MMA in older Americans. There are some concerns that increased folic acid intake as a result of folic acid fortification efforts in the US has adversely affected the vitamin B12 status [9]. Using the post-folic acid fortification data Selhub et al. [9] reported that high folate in vitamin B-12 deficient individuals negatively affected methlymalonyl CoA mutase and methionine synthase activities leading to increased MMA and total homocysteine (tHcy) concentrations, respectively. Also, Morris et al. reported [10] that high folate status in those with low vitamin B-12 status was related to anemia and cognitive impairment.

Previous reports on MMA concentrations were based on either small convenience sample or non-representative US population [11,12,13]. In this study, we used data reported in three nationally representative sample surveys, National Health and Nutrition Examination Surveys (NHANES) 1999–2000, 2001–2002 and 2003–2004 and derived population-based reference values. By combining three surveys into one analytic data, yielded a large sample size and increased the precision of the serum MMA estimate.

In this report, we presented reference values for serum MMA concentrations and the associations between serum MMA and sex, age, race-ethnicity, serum creatinine, a measure of kidney function and serum vitamin B-12 in the post-folic acid fortification period, 1999–2004 for US adolescents and adults.

## 2. Methods

### 2.1. Description of Survey and Study Sample

National Center for Health Statistics (NCHS) conducts National Health and Nutrition Examination Surveys (NHANES) on a periodic basis. NHANES was designed to be a complex, stratified, multi-stage probability sample survey. As part of the household interview, demographic, socioeconomic, dietary and health related data were collected in the participants’ home. A physician administered examination was conducted on household interviewed persons in Mobile Examination Centers (MEC). Also, blood and urine samples were collected in MEC for biochemical analyses. Certain populations such as young children, older persons, non-Hispanic black and Hispanic/Mexican were over sampled to yield reliable estimates for these groups. All NHANES protocols were approved by the NCHS Institutional Review Board and survey participants signed informed consent before participating in NHANES.

Beginning 1999, NHANESs were conducted as continuous, annual surveys rather than the periodic surveys. Briefly, NHANES 1999–2000 was conducted between March 1999 and December 2000 on 9965 individuals (all were home interviewed; 9282 were examined in MECs); NHANES 2001–2002 was conducted between January 2001 and December 2002 on 11,039 individuals (all were home interviewed; 10,477 were examined in MECs); and NHANES 2003–2004 was conducted between January 2003 and December 2004 on 12,761 individuals (10,122 were home interviewed; 9643 were examined in MECs). In this study, NHANES 1999–2000 [14], NHANES 2001–2002 [15] and NHANES 2003–2004 [16] were concatenated into one analytic database (NHANES 1999–2004) as per NCHS guidelines. A detailed description of these surveys has been reported earlier [17,18,19].

In the data analysis, individuals who reported their race-ethnicity as “unknown” or other than non-Hispanic white, non-Hispanic black, or Hispanic/Mexican were excluded due to small sample size. Also, individuals with missing values for sex, race-ethnicity, age, serum creatinine, serum vitamin B-12 and serum MMA were excluded from the data analysis. After applying the above exclusion criteria, the final study sample consisted of 18,569 aged ≥ 12 years. Of those, men were ≈49% (*n* = 9020), women were ≈51% (*n* = 9549), non-Hispanic white were ≈44% (*n* = 8170), non-Hispanic black were ≈23% (*n* = 4351), Hispanic/Mexican were ≈33% (*n* = 6048), adolescents (12 to <18 years) were ≈24% (*n* = 4546) and elderly persons (>60 years) were ≈23% (*n* = 4190). About 41% of the study participants reported use of supplements (Table 1).

### 2.2. Measurements

Data were collected on body measurements, demography, physical function, health condition, lifestyle behaviors, biochemical measurements of blood and urine and diet intake. Blood was collected from venipuncture in the MECs according to the standard protocols. MMA was measured in serum with gas chromatography/mass spectrophotometry. Vitamin B-12 was measured in serum with Quantaphase II radioassay kit (Biorad Laboratories, Hercules, CA, USA). Serum creatinine was measured using an autoanalyzer (Hitachi Model 917, Holliston, MA, USA, for NHANES 1999–2002; Beckman Synchron LX20, Brea, CA, USA, for NHANES 2003–2004). Detailed methodology is described elsewhere [20,21,22].

### 2.3. Statistical Analysis

As per NCHS’s guidelines, we used SUDAAN statistical software for Windows (SAS-Callable, version 10.0, Research Triangle Institute, Research Triangle Park, NC, USA) to account for complex survey design, differential probabilities of selection, non-coverage and non-response bias, sample weights, primary sampling units and stratification variables in the data analysis. Also, SAS (SAS for Windows, version 9.1, SAS Institute Inc., Cary, NC, USA) was used to manage and analyze data files.

In NHANES, 1999–2004, individuals whose age was ≥85 years were categorized as 85 years to protect the confidentiality of survey participants. This precluded us using the age as a continuous variable. In the data analysis, age was categorized into 19–30 years, 31–40 years, 41–50 years, 51–60 years, 61–70 years and >70 years old age groups. Standard errors were estimated with Taylor Linearization method because this procedure takes NHANES’s complex survey design into consideration. Cutoff values for 5th, 25th, 50th, 75th and 95th percentiles and arithmetic and geometric means for serum MMA concentrations were determined for sex, race-ethnicity, age, serum creatinine and serum vitamin B-12. Also, sex-, race-ethnicity-, age, serum creatinine- and serum vitamin B-12-specific serum MMA concentrations were determined with analysis of covariance. Because serum MMA concentrations were skewed, a natural logarithmic transformation was applied to satisfy the requirement for normality.

Additionally, we performed a sub group analysis on adult population (19 years or older) to determine the prevalence of serum MMA for <100 nmol/L, 100 ≤ 200 nmol/L, 200 ≤ 300 nmol/L and ≥300 nmol/L concentrations by race-ethnicity (non-Hispanic white, non-Hispanic black and Hispanic/Mexican) and age. Age was categorized into 19–30 years, 31–40 years, 41–50 years, 51–60 years, 61–70 and ≥71 years old groups. Statistical significance was set at *p* < 0.05 in all analyses.

## 3. Results

Population reference values (geometric means, medians and 5th to 95th percentiles) for serum MMA concentrations by sex, race-ethnicity, age, serum creatinine and serum vitamin B-12 for US population in the post-folic acid fortification period are presented in Table 2. Mean, median and 5th to 95th percentiles were higher for non-Hispanic white compared to non-Hispanic black, or Mexican American/Hispanic, higher for elderly persons compared to adolescents or young adults, higher for 4th quartile serum creatinine compared to 1st quartile serum creatinine category and higher for 1st quartile serum vitamin B-12 compared to 4th quartile serum vitamin B-12 category.

**Figure 1:**
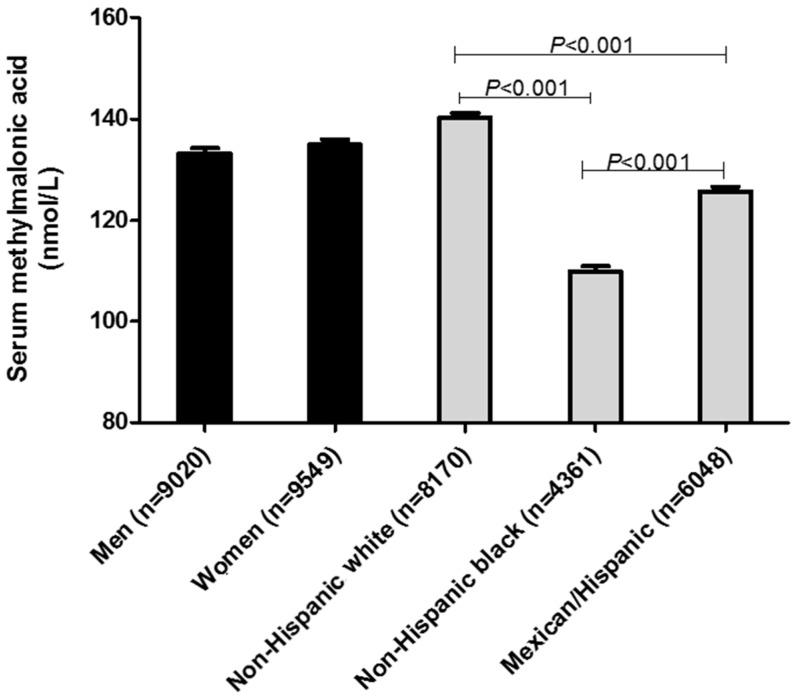
Adjusted sex and race-ethnicity-stratified geometric mean concentrations of serum methylmalonic acid (MMA) in ≥12 years old persons in the US (*n* = 18,569). National Health and Nutrition Examination Surveys 1999–2000, 2001–2002 and 2003–2004, conducted after the folic acid fortification commenced, were combined into one analytic data set, 1999–2004. Values for men and women were adjusted for race-ethnicity, age, supplement use, serum creatinine and serum vitamin B-12. Values for race-ethnicity were adjusted for sex, age, supplement use, serum creatinine and serum vitamin B-12. Adjusted serum MMA concentrations were not significantly different between men and women (*p* < 0.11). Adjusted serum MMA concentrations were significantly different between three races (*p* < 0.001). Analysis was performed on log serum MMA concentrations to satisfy normality.

**Figure 2:**
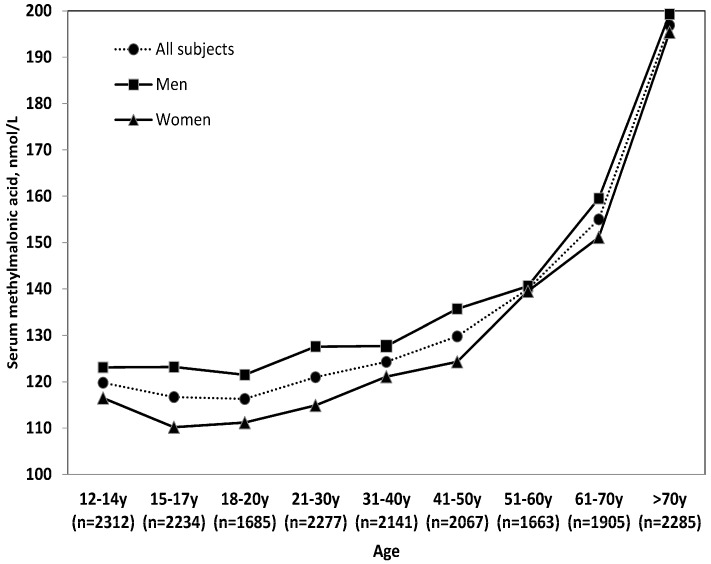
Age-stratified geometric mean concentrations of serum methylmalonic acid (MMA) for ≥12 years old persons in the US (*n* = 18,569). Values for age were adjusted for sex, race-ethnicity, supplement use, serum creatinine and serum vitamin B-12. Analysis was performed on log MMA concentrations to satisfy normality.

Sex- and race-ethnicity-specific and age-specific geometric mean MMA concentrations for US population in the post-folic acid fortification period are presented in Figure 1 and Figure 2, respectively. Geometric mean MMA concentration in non-Hispanic white (141.2 nmol/L) was ≈22.3% higher compared to non-Hispanic black (115.5 nmol/L) and was ≈18.5% higher compared to Hispanic/Mexican (119.2 nmol/L). Older adults (>70 years old) had ≈62.7% higher (196.9 nmol/L) geometric mean serum MMA compared to younger persons (21–30 years old) (121.0 nmol/L). In the adjusted analysis, race-ethnicity (*p* < 0.001) and age (*p* < 0.001) but not sex (*p* = 0.057) were significantly associated with serum MMA concentrations.

**Figure 3:**
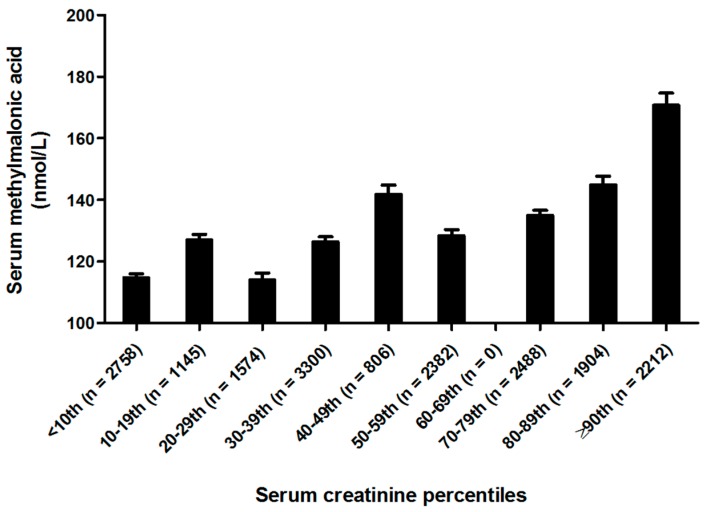
Serum creatinine stratified geometric mean concentrations of serum methylmalonic acid (MMA) for ≥12 years old persons in the US (*n* = 18,569). MMA values for serum creatinine concentrations were adjusted for sex, race-ethnicity, age, supplement use and serum vitamin B-12. Analysis was performed on log MMA concentrations to satisfy normality.

**Figure 4:**
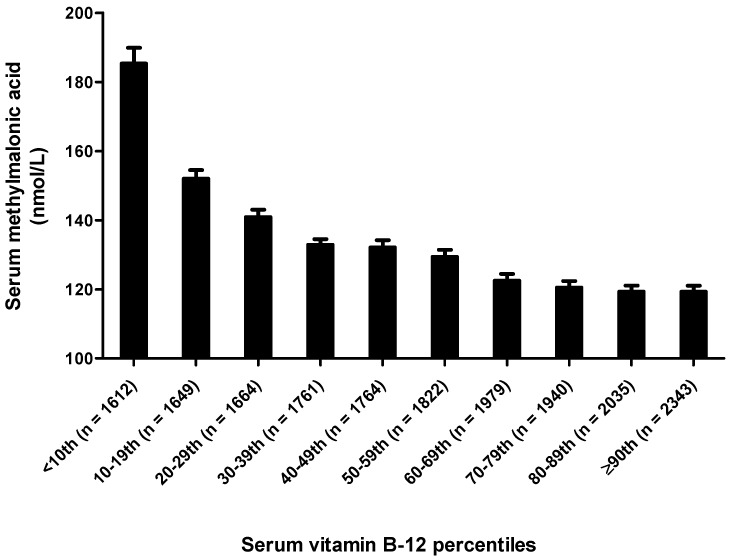
Serum vitamin B-12 stratified geometric mean concentrations of serum methylmalonic acid (MMA) for ≥12 years old persons in the US (*n* = 18,569). Serum MMA concentrations were adjusted for sex, race-ethnicity, age, supplement use and serum creatinine. Analysis was performed on log MMA concentrations to satisfy normality.

Serum MMA concentrations for US population according to serum creatinine concentrations and serum vitamin B12 concentrations in the post-folic acid fortification period are presented in Figure 3 and Figure 4, respectively. Not surprisingly, persons in the ≥90th percentile for serum creatinine concentrations had the highest serum MMA concentrations while the persons in the <10th percentile for serum creatinine had the lowest serum MMA concentrations. As expected, persons in the ≥90th percentile for serum vitamin B12 concentrations had the lowest serum MMA concentrations, while the persons in the <10th percentile for serum vitamin B12 had the highest serum MMA concentrations.

Prevalence (%) of serum MMA concentrations in non-Hispanic white, non-Hispanic black and Hispanic/Mexican by age (19 years or older) were presented in Table 3. Across all age groups, the prevalence of low serum MMA (<100 nmol/L) were lower in non-Hispanic white compared to non-Hispanic black, while the prevalence of high serum MMA (≥300 nmol/L) were higher in Hispanic white compared to non-Hispanic black. In non-Hispanic white in comparison with non-Hispanic black, the prevalence of high serum MMA (≥300 nmol/L) was almost double in 50 years and older age group. Whereas the prevalence for Hispanic/Mexican were either close to non-Hispanic white or in between non-Hispanic white and non-Hispanic black.

## 4. Discussion

In this study, we report the first data on reference values for serum MMA concentrations in the post-folic acid fortification period utilizing the data from 3 nationally representative sample surveys of US population (1999–2004). By combining NHANES 1999–2000, 2001–2003 and 2003–2004 cycles into one NHANES 1999–2004 database resulted in a large sample size (*n* = 18,579) and thus increased the precision of the serum MMA estimate. Because the data used in this study are based on the nationally representative sample, these results can be applied to the population reported in this study.

Circulating MMA concentrations are elevated in tissue vitamin B-12 deficiency. However, there are other factors which may affect serum MMA concentrations in humans such as kidney dysfunction, metabolism of odd chain fatty acids and amino acids such as methionine, isoleucine and threonine, intestinal bacteria [3] and genetic defects in methlymalonyl CoA mutase (*mut* 0 and *mut*-) [23]. It is unknown to what extent these can affect MMA balance in humans. In addition to MMA, compounds such as propionyl CoA and methyl citrate (formed from condensation of propionyl CoA and acetyl CoA by citrate synthase) are accumulated. It is well recognized that vitamin B-12 deficiency has been linked to neurological disorders such as myelopathy, neuropathy, depression and poor cognition [24,25,26]. The deleterious effects of vitamin B-12 deficiency on nervous system are attributed partly due to decreased methylation and partly due to deleterious effects of elevated MMA, propionly CoA, methylcitrate [1] and tHcy [27].

Serum vitamin B-12 is a major determinant of MMA concentrations. Several studies documented serum vitamin B-12 concentrations in the US population. In the Framingham study, 8%, 16% and 39% adults (26–83 years old) had plasma vitamin B-12 < 148, <185 and <258 pmol/L, respectively [28]. In the Framingham elderly population (67–96 years), 40.5% (222 out of 548) had serum vitamin B-12 < 258 pmol/L [11]. These studies were based on white population. In the Los Angeles elderly people (>60 years, *n* = 725), 11.8% had vitamin B-12 < 140 pmol/L and 16.6% had high MMA (>370–376 pmol/L) [13]. Morris et al. [8], using the data from the pre-folic acid fortification period (phase 2 of NHANES III) in 1145 older persons (age ≥ 65 years) reported ≈18% and ≈16% prevalence of serum high MMA (>370 nmol/L) in men and women, respectively. Thus, the existing data on the prevalence of vitamin B-12 deficiency in the US vary depending on the type of population studied and the criteria used to define the vitamin B-12 deficiency.

Serum MMA was generally stable at the age below 20 years but gradually increased afterward. The upward trend in serum MMA has been observed after the age 40 years. Geometric mean of serum MMA was ≈63% higher in elderly persons (>70 years) compared to those in the age group of 18–20 years. In a sub-group analysis, we found prevalence of high serum MMA in the older persons compared to the younger persons regardless of their race-ethnicity (Table 3). This age-related increase in serum MMA is caused by decreased kidney function [29] and increased prevalence of vitamin B-12 deficiency resulting from decreased vitamin B-12 absorption. The latter is caused by gastric atrophy accompanied by a decreased secretion of acid and pepsin [30,31,32] needed to release the protein-bound vitamin B-12 prior to its absorption. Use of medications by some elderly (e.g., as histamine H_2_ receptor antagonists and proton pump inhibitors) might lead to achlorhydria [33] prompting malabsorption of the protein-bound vitamin B-12 and thereby an elevated MMA.

Our data suggest that the race-ethnicity affect serum MMA concentration in the US. Non-Hispanic whites had significantly higher serum MMA concentrations compared to non-Hispanic black or Hispanic/Mexican in spite of higher use of vitamin/mineral supplements (56.7%) and a lower prevalence of the elevated serum creatinine (2.1%), a surrogate marker of kidney dysfunction. Additionally, we found by a sub-group analysis that non-Hispanic black have lower serum MMA concentrations compared to the other two race-ethnicities studied (Table 3). In non-Hispanic white compared to non-Hispanic black, the prevalence of high serum MMA (≥300 nmol/L) were ≈205%, ≈143% and ≈80% higher in 51–60 years old, 61–70 years old and >70 years old persons, respectively. Although the prevalence of kidney dysfunction is higher in non-Hispanic black compared to non-Hispanic white, the lower prevalence of high serum MMA in non-Hispanic black indicates that in this study kidney dysfunction is not a major factor in determining the serum MMA in older non-Hispanic black.

Recently Malloy et al. [34] reported that single nucleotide polymorphism in 3-hydroxyisobutyryl CoA hydrolase (HIBCH), is strongly associated with elevated MMA concentrations in Irish white population. This newly identified enzymatic polymorphism is more likely explanation for elevated MMA in whites compared to other race-ethnicities. This polymorphism influences the serum MMA concentrations independently of vitamin B-12 status [34]. Other variants in genes that do not encode vitamin B-12 metabolic genes (ASCF3, SUCLG1 and SUCLA) can also increase circulating MMA [35]. Additionally, it is possible that metabolic differences between race-ethnicities could contribute to the differences in serum MMA between races. Morris et al. [8] using the data from elderly persons reported high serum MMA in non-Hispanic white but the prevalence of high serum MMA in those subjects was similar to non-Hispanic black. In contrast, Carmel et al. [13] observed significantly lower vitamin B-12 concentrations in whites and Latin Americans compared to blacks. Nonetheless, the causes for differences between races regarding vitamin B-12 metabolism needs further attention.

We found serum MMA concentrations increased with age especially after the age of 51–60 years. Similarly, Risch et al. [36] in Swiss population (*n* = 1143) reported significant increment in MMA along with increments of tHcy although vitamin B-12 and holoTC remained steady after the age 60 years. This increased MMA in elderly persons can be attributed to impaired kidney function. In elderly subjects (>71 years old), the metabolic vitamin B-12 deficiency was 30% compared to only 12% in younger subjects (<50 years old) who were attending a stroke prevention clinic [37]. Although a direct relation between serum creatinine, a surrogate marker of kidney function and serum MMA concentrations exists, all cases of high MMA cannot be attributed to renal dysfunction in older adults. It has been hypothesized that elevated MMA may damage the kidney [38]. It is not known whether increased MMA causes impaired kidney function or impaired kidney function leads to elevated MMA concentrations.

In comparison to pre-folic acid fortification period [39], we report elevated MMA in the post-folic acid fortification period in all subjects (pre, 137 nmol/L vs. post, 155 nmol/L), men (pre, 141 nmol/L vs. post, 157.2 nmol/L), women (pre, 133 nmol/L vs. post, 152.9 nmol/L). This further strengthens the notion that increased folic acid intake in the post folic acid fortification period may have negatively affected the vitamin B-12 status leading to increased circulating MMA concentrations. The possible explanation for this phenomenon is that the high folate status in the post-folic acid fortification period may have adversely affected the methylmalonyl CoA mutase enzyme leading to elevated MMA [9]. Other unknown causes may have also contributed to elevated serum MMA in the post-fortification period.

To date, there is no consensus defining a low vitamin B-12 status based on serum vitamin B-12 and MMA, because each marker has insufficient diagnostic specificity and sensitivity [40]. In some cases, serum vitamin B-12 does not reflect vitamin B-12 nutritional status because only ≈6–20% is bound to holoTC, which is a biologically active vitamin B-12 fraction, capable of delivering vitamin B-12 to tissues whereas the remaining majority is bound to haptocorrin [41]. Recently, measurement of holoTC has been suggested as a sensitive and early marker of vitamin B-12 deficiency [42]. However, in a recent review by Golding [43] provided the evidence that holoTC concentration cannot be used as a reliable and a sensitive marker of early vitamin B-12 deficiency any more than serum vitamin B-12 concentration because this concept is based on a faulty model for the diagnosis and staged development of vitamin B-12 deficiency proposed by Herbert in 1980s. Further Golding [44] reported that holoTC is much less sensitive than circulating MMA concentration. This fact is further corroborated by Remacha et al. [45] in a study where holoTC concentrations were not decreased in 1/3 of the subjects in spite of low vitamin B-12. In fact, holoTC concentrations were significantly reduced in 48% folate deficient individuals and this decrease was not due to vitamin B-12 deficiency suggesting a role for folate in holoTC metabolism.

Although MMA appears to be a sensitive marker of vitamin B-12 deficiency, MMA concentrations are elevated in some subjects with kidney dysfunction [29] and in patients with polymorphisms [34]. Like MMA, holoTC is also elevated in renal dysfunction [46] due to the decreased uptake by erythroid cells. In contrast, decreased holoTC in serum could be the result of increased uptake of vitamin B-12 by erythroid cells [45]. Elevated tHcy is not specific to vitamin B-12 deficiency. Thus, there is no single universal standard for the measurement of vitamin B-12 deficiency. Therefore, a multiple marker approach has been proposed for the diagnosis of vitamin B-12 deficiency. Hermann et al. [46] proposed a two-marker approach (serum holoTC and MMA concentrations). Recently, Fedosov et al. [47] derived a novel systematic algorithm {(vitamin B-12 status) = log10[(holoTC × B12)/(MMA × Hcy)]–(age factor)} for diagnosis of vitamin B-12 deficiency using four markers of vitamin B-12 status (serum vitamin B-12, holoTC, MMA and tHcy concentrations). They proposed five grades of vitamin B-12 status, i.e., elevated vitamin B-12, vitamin B-12 adequacy, low B-12, possible vitamin B-12 deficiency and probable vitamin B-12 deficiency based on the circulating concentrations of four biomarkers of vitamin B-12 status. However, Fedosov et al.’s [47] approach is not applicable to children below 18 years of age and pregnant women.

## 5. Conclusions

In this large population study based on the data collected in the post-folic acid fortification era, age, race-ethnicity, serum creatinine and serum vitamin B-12 are related to serum MMA concentrations. Based on serum MMA concentrations across the age groups, serum MMA concentrations begin to rise after the age 40 years, continue to rise afterwards and then steeply increase after the age 70 years. Serum MMA utility as a gold standard for vitamin B-12 deficiency is limited in patients with normal renal function and in younger persons.

## Figures and Tables

**Table 1:** Characteristics of study population: National Health and Nutrition Examination Survey (NHANES), 1999–2004 ^1^.

Characteristic	Men	Women	*p*-Value ^2^
	(*n* = 9020)	(*n* = 9549)	
Race-ethnicity			
Non-Hispanic white, *n*, (%)	3971 (48.9)	4199 (51.1)	0.023
Non-Hispanic black, *n*, (%)	2140 (45.2)	2211 (54.8)	<0.0001
Hispanic/Mexican, *n*, (%)	2909 (49.7)	3139 (50.3)	0.72
Age (year)			
12–14, *n* (%)	1125 (51.1)	1187 (48.9)	0.41
15–17 years, *n* (%)	1178 (51.4)	1056 (48.6)	0.30
18–40 years, *n* (%)	2775 (49.7)	3328 (50.3)	0.63
41–65 years, *n* (%)	2254 (49.1)	2274 (50.9)	0.16
>65 years, *n* (%)	1590 (42.6)	1598 (57.4)	<0.0001
Supplements use ^3^			
Yes, *n* (%)	3222 (42.9)	4459 (57.1)	<0.0001
No, *n* (%)	5798 (54.3)	5090 (45.7)	<0.0001
Serum creatinine, μmol/L (mean ± SE)	83.4 ± 0.5	64.7 ± 0.4	<0.001
Serum vitamin B-12, pmol/L (mean ± SE)	380.8 ± 7.0	418.5 ± 17.1	0.04

^1^ NHANES 1999–2000, 2001–2002 and 2003–2004, conducted after the folic acid fortification commenced, were combined into one analytic data set, 1999–2004. *n* = 18,569. Weighted *n* = 204,316,278. Percentages in parentheses are based on weighted sample size. ^2^ Significance between men and women. Rao-Scott χ^2^ test or unpaired *t*-test. ^3^ Persons who took vitamin/mineral supplements one month prior to the survey.

**Table 2:** Population reference values for serum methylmalonic acid (MMA) concentrations in the US population: data from the National Health and Nutrition Examination Survey, 1999–2004 ^1^.

	*n*	Mean ± SE ^2^	Percentiles				
			5th	25th ^3^	50th	75th	95th
		MMA, nmol/L					
All subjects	18,569	155.0 ± 2.1	69.6	99.4	129.3	169.4	297.0
Sex							
Men	9020	157.2 ± 2.6	70.0	100.0	129.7	169.8	299.2
Women	9549	152.9 ± 2.9	69.4	97.4	125.6	167.7	289.7
Race-ethnicity							
Non-Hispanic white	8170	162.1 ± 2.4	79.0	106.3	134.4	179.1	300.0
Non-Hispanic black	4351	133.3 ± 8.1	59.8	83.5	109.1	139.5	229.9
Hispanic/Mexican	6048	135.7 ± 2.9	60.0	89.4	109.9	149.5	269.8
Supplement use ^4^							
Yes	7681	155.3 ± 3.2	69.8	99.5	129.4	169.2	281.4
No	10,888	154.6 ± 2.4	69.4	99.3	129.2	169.7	301.9
Age (year)							
12–14	2312	130.0 ± 2.6	69.5	89.9	116.8	149.4	226.1
15–17	2234	135.8 ± 6.0	65.9	89.3	109.8	139.9	227.3
18–40	6103	134.1 ± 1.7	69.0	89.8	119.2	150.0	248.7
41–65	4732	152.4 ± 2.8	73.3	100.3	129.6	169.5	274.8
>65	3188	236.1 ± 9.4	89.8	129.6	169.4	229.9	476.4
Serum creatinine ^5^ (µmol/L)							
<55.96	5477	132.9 ± 1.7	59.9	89.3	109.8	149.3	254.7
55.96 ≤ 70.71	4106	146.4 ± 2.7	69.5	99.0	128.3	159.9	262.2
7.71 ≤ 79.8	4870	147.1 ± 2.7	72.3	99.7	129.0	160.1	272.6
≥79.8	4116	190.6 ± 6.3	79.6	112.5	149.1	199.6	377.7
Serum vitamin B-12 ^5^ (pmol/L)							
<260.96	4087	202.3 ± 6.1	79.4	110.0	149.7	209.7	428.0
260.96 ≤ 340.94	4363	146.6 ± 1.6	69.9	99.9	129.7	169.4	269.4
340.94 ≤ 445.91	4754	138.3 ± 2.9	69.4	97.6	119.7	152.0	240.0
≥445.91	5365	133.0 ± 2.5	68.1	89.7	116.5	149.3	232.5

^1^ Same as Table 1. ^2^ Values are geometric means ± standard error of geometric means. ^3^ Medians. ^4^ Persons who took vitamin/mineral supplements one month prior to the survey. ^5^ Categorized into quartiles.

**Table 3:** Prevalence (%) of serum methylmalonic acid (MMA) concentrations in non-Hispanic white, non-Hispanic black and Hispanic/Mexican by age: National Health and Nutrition Examination Survey, 1999–2004 ^1,2,3^.

Age, Year	≤100 nmol/L			100 ≤ 200 nmol/L			200 ≤ 300 nmol/L			≥300 nmol/L		
	NW	NB	H/M	NW	NB	H/M	NW	NB	H/M	NW	NB	H/M
19–30 (*n* = 2948)	26.8	50.0	40.2	59.6	47.0	50.8	10.8	2.3	6.0	2.9	0.8	3.0
31–40 (*n* = 2008)	23.9	43.5	40.0	64.1	48.7	51.0	8.9	5.4	6.0	3.2	2.4	2.9
41–50 (*n* = 1929)	19.2	36.8	34.7	66.2	55.2	53.3	10.8	5.6	8.5	3.8	2.5	3.5
51–60 (*n* = 1516)	12.1	28.3	26.6	67.0	61.2	61.0	15.4	8.7	7.3	5.5	1.8	5.1
61–70 (*n* = 1684)	9.5	24.6	19.9	64.8	61.2	62.2	16.7	10.5	11.5	9.0	3.7	6.5
>70 (*n* = 2069)	3.9	4.8	8.9	50.3	56.2	55.1	27.1	28.5	17.5	18.7	10.4	18.6

^1^ Same as Table 1. *n* = 12,154. Sub-group analysis was performed on adult population only (age 19 years or older). ^2^ Abbreviations: NW, non-Hispanic white; NB, non-Hispanic black; H/M, Hispanic/Mexican. ^3^ Prevalence were age-specific and race-specific.
